# Biomechanical determinants of endothelial permeability assessed in standard and modified hollow-fibre bioreactors

**DOI:** 10.1098/rsif.2023.0222

**Published:** 2023-08-23

**Authors:** Stephen G. Gray, Peter D. Weinberg

**Affiliations:** Department of Bioengineering, Imperial College London, London SW7 2AZ, UK

**Keywords:** wall shear stress, haemodynamics, macromolecule, thrombin, L-NAME, epithelium

## Abstract

Effects of mechanical stress on the permeability of vascular endothelium are important to normal physiology and in the development of atherosclerosis. Here we elucidate novel effects using commercially available and modified hollow-fibre bioreactors, in which endothelial cells form confluent monolayers lining plastic capillaries with porous walls, contained in a cartridge. The capillaries were perfused with a near-aortic waveform, and permeability was assessed by the movement of rhodamine-labelled albumin from the intracapillary to the extracapillary space. Permeability was increased by acute application of shear stress and decreased by chronic shear stress compared with a static control: this has previously been shown only for multidirectional flows. Increasing viscosity reduced permeability under both acute and chronic shear; since shear rate remained unchanged, these effects resulted from altered shear stress. Reducing pulsatility increased permeability, contrary to the widely held assumption that flow which is highly oscillatory causes endothelial dysfunction. Chronic convection across the monolayer increased effective permeability more than could be explained by the addition of advective transport, contrary to results from previous acute experiments. The off-the-shelf and modified bioreactors provide an excellent tool for investigating the biomechanics of endothelial permeability and have revealed novel effects of flow duration, viscosity, pulsatility and transmural flow.

## 1. Introduction

Transport of macromolecules across vascular endothelium is essential for normal physiological functions such as the delivery of lipids, metal ions, hormones and other signalling molecules to cells. Abnormal transport can be important in pathological processes. A case of particular interest is atherosclerosis, which is triggered by elevated transendothelial transport of low-density lipoprotein. The patchy distribution of atherosclerosis within the arterial tree has been attributed to influences of spatially varying mechanical stresses on endothelial permeability [1–4].

Assessment of patterns of mechanical stresses and permeability *in vivo* can only determine correlations between the two. Establishing causality and mechanisms requires intervention. Previous work has modified mechanical stress—principally haemodynamic wall shear stress (WSS)—by ligating vessels *in vivo* [5,6] or by artificial perfusion of vessels *in situ* or *ex vivo* [7,8]. Fewer such studies have been conducted than their potential pathological importance would merit, reflecting the substantial technical difficulties.

Many studies have instead investigated the link by measuring permeability while applying stresses to endothelial monolayers *in vitro*. Methods include culturing cells on porous microcarrier beads in a column [9], on a porous membrane under a rotating disc [10], and in wells swirled on an orbital shaker or in microchannels, both in combination with tracers that bind to the substrate under the cells [11,12]. Effects of pressure-driven transendothelial flow rather than luminal flow have been studied using porous membranes in a custom apparatus [13].

Here we evaluate and use the hollow-fibre bioreactor. The apparatus, introduced by Knazek *et al*. [14], consists of multiple plastic capillary tubes (hollow fibres) with porous walls, encased in a cartridge. Manifolds at each end of the cartridge allow perfusion of the capillaries via an external circuit that includes a pump and oxygenator; the whole apparatus is maintained in a cell culture incubator. Such devices are available commercially. Growing cells in the extracapillary space of the cartridge mimics tissue architecture and allows the collection of cell-secreted products. The high surface area-to-volume ratio and porosity of the capillary walls gives excellent solute transport [15]. Alternatively, endothelial cells [16] and epithelial cells [17] can be grown on the *inside* of the capillaries, forming confluent monolayers on the luminal surface of the wall. Introducing tracer into the intracapillary space and monitoring its emergence into the extracapillary space allows the measurement of permeability in monolayers exposed to shear.

The system has been used to determine effects on solute permeability of co-culture with other cell types [16,18,19] or of conditioned medium [19]. It has also been used to determine whether transport is receptor mediated or not [20], and to assess transendothelial movement of cells [21] as well as solutes. Finally, it has been used to examine influences of different shear levels on biological properties such as protein secretion [22] or proliferation [23], but we are not aware of any previous studies that employed hollow-fibre bioreactors to examine effects of different mechanical stresses on endothelial permeability to solutes. Here we use standard and modified bioreactors for that purpose, generating novel findings on the influences of viscosity, flow waveform and transmural flow.

## 2. Methods

### 2.1. Isolation and characterization of porcine aortic endothelial cells

Descending thoracic aortas of Landrace Cross pigs aged four–six months were obtained at abattoir and stored in Hanks balanced salt solution (HBSS) containing penicillin (200 U ml^−1^), streptomycin (200 µg ml^−1^), amphotericin (5 µg ml^−1^) and gentamycin (100 µg ml^−1^) for up to 24 h. Porcine aortic endothelial cells (PAECs) were isolated by perfusing the lumen with collagenase as previously described [24]. They were then resuspended in Dulbecco's modified eagle medium (DMEM, Sigma-Aldrich) supplemented with 20% fetal bovine serum (FBS), penicillin (100 U ml^−1^), streptomycin (100 µg ml^−1^), amphotericin (2.5 µg ml^−1^), gentamycin (50 µg ml^−1^), L-glutamine (5 mM) and endothelial cell growth factor (ECGF, 5 µg ml^−1^, Sigma-Aldrich), henceforth termed ‘DMEM with supplements’. Cells were cultured under 5% CO_2_ at 37°C. Medium was changed after 1 h to reduce smooth muscle cell contamination, after 24 h and then every 2 days. Cells were used at passages 1–3.

Endothelial cell purity was assessed from the internalization of Dil-labelled acetylated LDL (Molecular Probes), which was added to confluent monolayers at a final concentration of 10 µg ml^−1^ and incubated at 37°C for 12 h. After fixation in 4% paraformaldehyde and nuclear staining with DRAQ5 (BioStatus, 1 : 1000), the cells were imaged using a laser scanning confocal microscope (Leica SP5) with a 10× 0.40NA objective. Dil-acetylated-LDL was excited at 514 nm and detected between 539 and 593 nm; equivalent wavelengths for DRAQ5 were 633 and 675–725 nm. The fraction of cells taking up Dil-acetylated-LDL was determined with a manual counting program developed in MATLAB (The MathWorks, Inc).

### 2.2. Bioreactor characteristics, activation and coating

**Figure 1.**
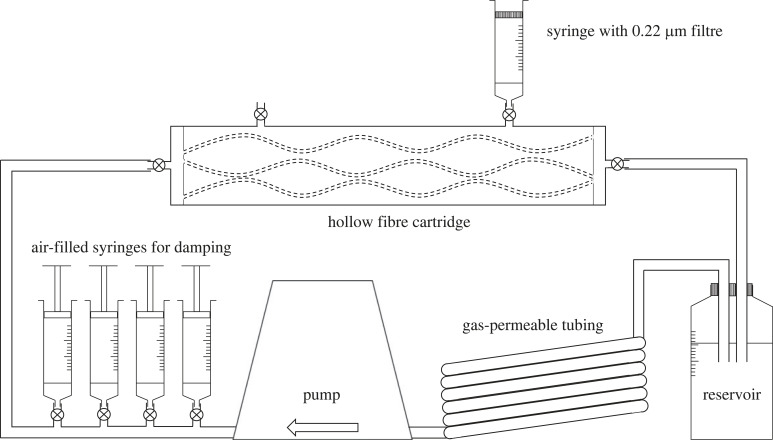
Diagram of the hollow-fibre bioreactor. Air-filled syringes for damping and the syringe with a 0.22 µm filter are modifications of the commercial apparatus and were used only in experiments assessing permeability under damped pulsatile flow and transendothelial flow, respectively. For experiments under static conditions, fluid was circulated in the extracapillary space rather than the intracapillary space by connecting the tubing to the two ports on the top of the cartridge. For clarity, only 3 of 20 capillary fibres are shown within the cartridge. The entire apparatus is mounted on a stand (not shown) that allows it to be transferred between incubator and hood.

The hollow-fibre bioreactor cartridges contained 20 ‘Polysulfone Plus’ capillaries, each approximately 10 cm long with 700 µm inner diameter, wall thickness 300 µm and pore size 0.1 µm. Total surface area was 70 cm^2^, supporting approximately 10^7^ cells. Flow was provided by a reservoir of medium connected to a positive displacement (Duet) pump in a closed circuit, and gas exchange was enabled by a coil of gas-permeable tubing (figure 1). All components were purchased from FiberCell Systems Inc., MD, USA.

Cartridges, connectors and the reservoir were sterilized with UV light prior to cell seeding. The fibres were then perfused with 70% ethanol for 1 min and 5% gelatin for 10 min. The cartridge was rotated 180° and left for a further 10 min before being placed in the incubator for 1 h, during which time the cartridge was rotated 180° every 15 min to distribute the gelatin more evenly. The hollow fibres were flushed with phosphate-buffered saline (PBS) and the reservoir was then attached to the circuit. Then 200 ml of DMEM with supplements was pumped through the gas-permeable tubing at a flow rate of 15 ml min^−1^ for 2 h prior to cell seeding.

### 2.3. Seeding the bioreactor

A confluent T75 flask of PAECs was trypsinized and the solution centrifuged at 220×*g* for 5 min. The pellet was resuspended in 5 ml of DMEM with supplements and the cells counted (normally 5–8 × 10^6^ cells). In a laminar flow hood, the cell suspension was slowly introduced into the intracapillary space, while the extra volume of fluid this introduced was allowed to permeate the fibre walls and leave from the extracapillary space.

The bioreactor was returned to the incubator for 1 h and the cartridge rotated 180° every 10 min to distribute the cells more evenly. The bioreactor was then removed from the incubator and fresh medium slowly flushed through the intracapillary space. Loose cells collected in the flushing solution were counted to determine the seeding efficiency (normally 40–60%). The seeding procedure was then repeated using a second T75 flask to reduce the time required to achieve confluence. The efficiency of the second seeding was approximately 40–50%.

The pump and gas-permeable tubing were attached to the extracapillary circuit, the flow rate set to 5 ml min^−1^, and the bioreactor returned to the incubator for 48 h, with medium in the intracapillary and extracapillary circuits being changed every 24 h. The pump and gas-permeable tubing were then attached to the intracapillary circuit; the flow rate of 5 ml min^−1^ was maintained for 24 h and then increased to 15 ml min^−1^. The pump in combination with its system of one-way valves gave a pulsatile flow waveform.

### 2.4. Assessing endothelial cell confluence

Glucose concentrations were monitored to determine when the monolayer had reached confluence; glucose consumption is reduced on contact inhibition and quiescence [25]. Samples of medium were analysed using glucose detection strips and a GluCell meter.

The permeability of the monolayer to rhodamine-labelled albumin was measured as described below soon after seeding and at several times after glucose measurements indicated a quiescent monolayer, to determine whether permeability had reached a steady, low level.

To image the monolayers after permeability experiments had been completed, PAECs were fixed *in situ* for 1 h with 15% paraformaldehyde. The bioreactor was opened with a pipe cutter; individual fibres were then removed from the cartridge and sectioned longitudinally with a razor blade and custom jig. Sectioned fibres were dehydrated in ascending concentrations of ethanol. The ethanol was replaced with CO_2_ by critical point drying. Samples were then placed on conducting carbon pads on aluminium stubs, coated with gold and imaged by scanning electron microscopy (SEM) using a JEOL JSM 5610 LV microscope with an accelerating voltage of 20 kV and emission current of 10 µA.

### 2.5. Preparation of tracer

Detailed methods are given elsewhere [26]. Briefly, bovine serum albumin (BSA) was mixed with sulforhodamine B acid chloride, and then purified of free dye and suspended in a 1 : 10 dilution of Tyrode's salt solution in water by gel filtration. The conjugate was lyophilized and stored at −20°C. Before use, it was reconstituted in water to 1/10th of its original volume, further purified with neutralized activated charcoal (0.35 g g^−1^ of protein) and sterile filtered.

### 2.6. Measuring monolayer permeability

The serum content of the culture medium in the reservoir was reduced from 20% to 10% following 10 days of culture and reduced to 4% 24 h before each permeability experiment. For the tracer experiment itself, the solution in the reservoir and gas-permeable tubing was replaced with DMEM, supplements other than FBS, 4% BSA and 1 mg ml^−1^ rhodamine-labelled albumin. The tracer solution was circulated for approximately 1 h in all experiments with flow. For static studies, tracer was not circulated but simply added to the intracapillary space using syringes and left there for 1 h. Samples were taken from intracapillary and extracapillary compartments, and tracer fluorescence in them was measured using a fluorimeter (model 6285, Jenway) with excitation and emission wavelengths of 570 and 600 nm, respectively. The concentration of rhodamine albumin in each diluted sample was determined from a standard curve.

Permeability (*P*) is defined as the flux of solute (*J*_s_) across a known surface area of membrane (*S*) per unit concentration difference across the membrane (Δ*C*)

$$P=\frac{J_{s}}{\left(S\times \Delta C\right)}.$$

The term is generally used to refer to diffusive transport, but a vesicular component cannot be ruled out in these experiments (see below). *J_s_* was determined from the concentration of rhodamine albumin in the extracapillary space (CECS), the volume of the extracapillary space (VECS), and the time after the addition of the tracer to the intracapillary space (*t*, normally 3600 s)

$$J_{s}=\frac{\left(\mathrm{CECS}\times \mathrm{VECS}\right)}{t}.$$

The low concentration of tracer in the extracapillary space was ignored when calculating the permeability from *J*_s_. This assumption means that baseline permeabilities will be underestimated by approximately 10%.

The resistances of the fibre wall without cells (*R*_fibre_) and of the fibre wall with the cell monolayer (*R*_total_) were calculated as the reciprocals of the corresponding permeabilities, *P*_fibre_ and *P*_total_. (The average value of *P*_fibre_ was 5.05 × 10^−06^ cm s^−1^.) *R*_fibre_ was subtracted from *R*_total_ to give the resistance of the cell monolayer alone (*R*_endothelium_), and its reciprocal gave the permeability of the monolayer (*P*_endothelium_)

$$\begin{array}{c} \;\\ P_{\mathrm{endothelium}}=\frac{1}{\left(R_{\mathrm{total}}-R_{\mathrm{fibre}}\right)},\; \end{array}$$

where

$$\begin{array}{rl} & R_{\mathrm{total}}=\frac{1}{P_{\mathrm{total}}}\\ & R_{\mathrm{fibre}}=\frac{1}{P_{\mathrm{fibre}}}. \end{array}$$

Several permeability measurements under different conditions were conducted in each bioreactor. Experiments ended 12–14 days after the first permeability experiment.

### 2.7. Effect of thrombin and L-NAME on endothelial permeability

In the first series of experiments, agents known to affect endothelial permeability were added to the system to determine whether the expected endothelial response could be detected. An absence of effect might indicate that the permeability was dominated by the resistance of the fibre wall, for example as a result of a failure to form a confluent monolayer with tight junctions. The experiments were carried out under continuous pulsatile flow, termed ‘chronic shear stress' (CSS; see next section). Thrombin (Sigma-Aldrich; 10 U ml^−1^) was added for a period of 1 h before permeability was measured. Production of NO was inhibited by the addition of the L-arginine analogue N^ω^-nitro-l-arginine methyl ester (L-NAME; Sigma-Aldrich; 500 µM) for 24 h prior to the permeability measurement.

### 2.8. Permeability under chronic shear stress, acute shear stress and static conditions

In the second series of experiments, the PAECs within the bioreactor were exposed to different durations of flow or to no flow. Following the initial permeability measurement under CSS, a further measurement was made after exposure to static conditions for 2–4 days, then after an acute application of shear stress (ASS) and finally after a repeat of the initial CSS condition. This procedure was repeated two–three times per bioreactor.

The flow rate of 15 ml min^−1^ gives a WSS of 0.375 Pa according to the manufacturer. The flow rate was confirmed by use of a transit-time ultrasound flow probe (Transonics) coupled to an analogue-to-digital converter and data analysis system (Notochord). Flow was maintained for 3–10 days at a time for CSS, depending on the interchanges between experimental conditions, and for 4 h after static conditions for ASS.

For static conditions, syringes were filled with DMEM and supplements and attached to the intracapillary space, so that the fibres remained full but not perfused, while the pump and gas-permeable tubing were used to perfuse the extracapillary space. Thus cells on the fibres acquired nutrients and oxygen, and had waste products removed, but were shielded by the fibre wall from the direct influence of flow. This method has been used by Milovanova *et al.* in their study of effects of shear on endothelial proliferation [23]; they demonstrated there was no change in oxygen delivery to the endothelial cells compared with intracapillary perfusion. Flow was maintained at 5 ml min^−1^ for approximately 3–5 days prior to permeability measurement. Note that mixing effects of the flow will have had a negligible influence on transport because the concentration of tracer was always much lower in the extracapillary than the intracapillary space.

### 2.9. Permeability with increased viscosity

Experiments were conducted in new bioreactors using medium that had been modified to obtain rheological properties analogous to those of blood. Following the technique outlined by van den Broek *et al*. [27], xanthan gum (Sigma-Aldrich, 0.69 g l^−1^) was UV sterilized, added slowly to DMEM plus supplements and stirred for 24 h to create a medium with viscosity of 4 × 10^−3^ Pa s. The sequence of CSS, static and ASS experiments was then repeated.

### 2.10. Permeability in modified bioreactors

A third series of experiments compared the normal pulsatile flow (CSS) with chronic flow in which almost all the pulsatility had been damped, and also compared it with CSS plus transmural flow. These experiments required modification of the bioreactor. In order to damp the pulsations, four 50 ml syringes filled with air were connected to the flow circuit (figure 1). Damping was confirmed with the transit-time flow meter.

For the transmural flow experiments, one of the ports accessing the extracapillary space was opened and a syringe with a 0.22 μm filter replacing the plunger was added to the port to expose the system to atmospheric pressure (figure 1). This modification created a pressure difference across the capillary wall and permitted transmural flow while maintaining sterility. After several hours, transmural flow across the capillary walls and into the cartridge and syringe stabilized to approximately 0.1 ml h^−1^. The syringe was replaced every few hours as the rising level of medium in it would otherwise have significantly altered the transmural pressure difference. All fluid entering the extracapillary space and the attached syringe was collected; its volume and its tracer concentration were measured to permit calculation of permeability.

All pulsatile, damped pulsatile and transmural flow experiments were carried out for approximately 3–5 days prior to the measurement of transport.

### 2.11. Data analysis

The statistical significance of differences was calculated using Student's *t*-test or ANOVA within each group of experiments and differences were deemed significant if *p* < 0.05. There was no significant difference between bioreactors within each experimental series, so all bioreactors were treated as equal: pooled experimental data were compared with pooled control data from the same series. The following symbols are used in histograms: *, *p* < 0.05; **, *p* < 0.01; ***, *p* < 0.001.

## 3. Results

### 3.1. Assessment of endothelial purity and confluence

The fraction of cells taking up Dil-acetylated-LDL was 99.7% (s.e.m. 0.07%; *n* = 5 isolations).

**Figure 2.**
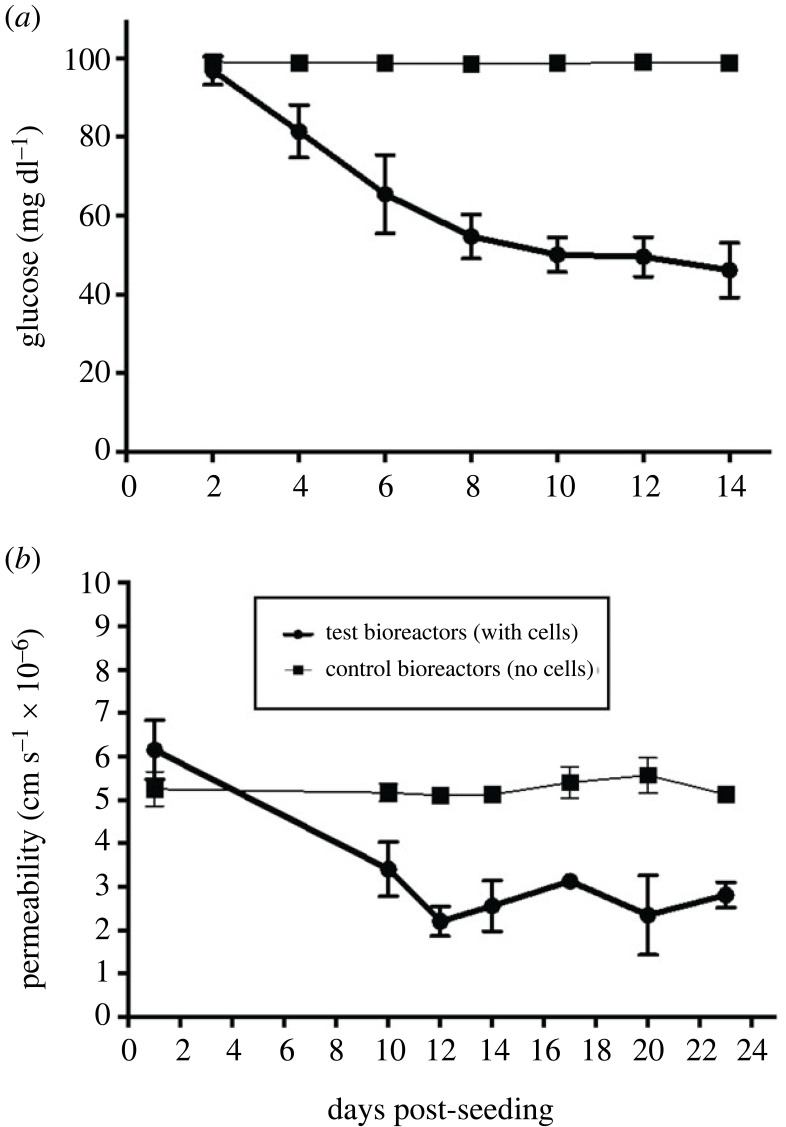
Glucose concentration and permeability as a function of time after seeding. (*a*) Glucose concentration in the medium was measured every second day for all bioreactors (*n* = 27). (*b*) Permeability of the endothelium (*P*_endothelium_) and permeability of the fibres (*P*_fibre_) were measured in four bioreactors with cells and four without cells, respectively. Mean ± s.e.m. Note the different *x*-axis scales.

Glucose concentration in the medium is shown as a function of time after seeding in figure 2*a*. Cells were exposed to chronic shear stress (CSS). As a control, concentrations were also measured in cell-free bioreactors.

We interpret the data as follows. PAECs reached confluence within 2–3 days after seeding. This led to a steady consumption of glucose for approximately the next 4 days, after which glucose consumption reduced and then reached a new but lower steady level, as expected for quiescent endothelium, at approximately 10 days.

Medium was changed when glucose was depleted by approximately 50%, which took between 10 and 16 days in different bioreactors. CSS was maintained.

Figure 2*b* shows permeability to rhodamine-labelled albumin as a function of time after seeding in four bioreactors. Permeability was also measured at equivalent times in cell-free bioreactors. Permeability in experimental and control bioreactors was essentially identical at day 1. Subsequent measurements in seeded bioreactors averaged less than half of those in control bioreactors and their level was approximately constant from day 10 onwards. This is consistent with a tight barrier having been formed by the time that glucose levels had depleted by 50% and the medium was changed. Permeability measurements reported below were made only after this point.

**Figure 3.**
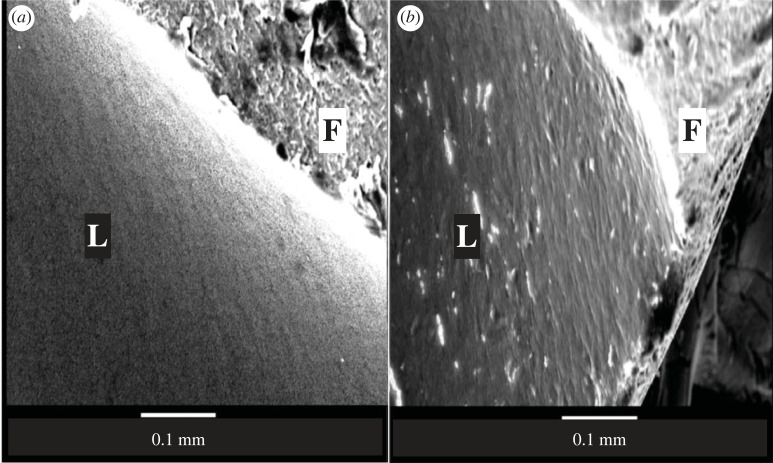
Scanning electron micrographs showing a confluent monolayer on a hollow fibre. SEM images show the luminal surface of (*a*) a fibre without cells and (*b*) a fibre with cells after a prolonged experiment. L = luminal surface, F = cut edge of fibre. Images acquired at ×250 magnification.

Figure 3 shows an *en face* SEM image of the luminal surface of the fibre and of an endothelial monolayer on the fibre at the end of a series of experiments. Contiguous endothelial cells and continuous boundaries are evident in the latter and the distinctive appearance of the unseeded fibre is not visible at any location or magnification, demonstrating that repeated permeability measurements and changes in flow do not substantially disrupt the confluent monolayer established soon after seeding. A larger area, again indicating a confluent monolayer with intact intercellular junctions, is shown in figure S1 of the electronic supplementary material.

### 3.2. Effects of thrombin and L-NAME

**Figure 4.**
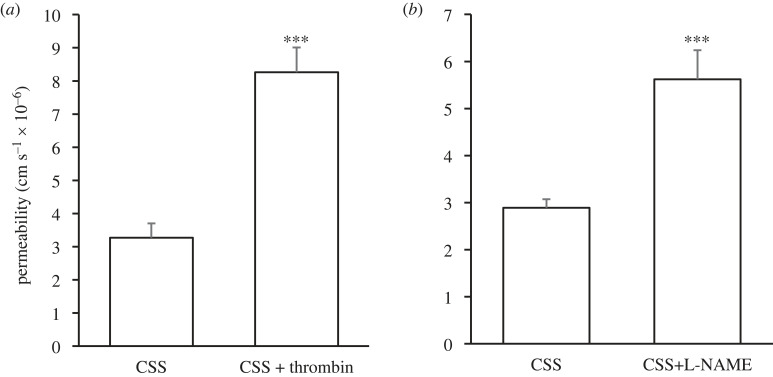
Effect of thrombin and L-NAME on monolayer permeability under chronic shear stress. (*a*) Permeability under CSS without thrombin, and after thrombin (10 U ml^−1^) was allowed to circulate for 1 h under CSS. Thrombin significantly increased permeability. (*b*) Permeability under CSS without L-NAME, and after L-NAME (500 µM) was allowed to circulate for 24 h under CSS. L-NAME significantly increased permeability. Mean + s.e.m.

Thrombin significantly increased permeability (8.26 ± 0.75 × 10^−6^ cm s^−1^; *p* < 0.0001; *n* = 9) compared with CSS without thrombin (3.27 ± 0.43 × 10^−6^ cm s^−1^; *n* = 10) (figure 4*a*).

Permeability with L-NAME (5.62 ± 0.62 × 10^−6^ cm s^−1^; *p* < 0.0001; *n* = 9) was also significantly higher than permeability for controls (2.89 ± 0.18 × 10^−6^ cm s^−1^; *n* = 9) (figure 4*b*).

### 3.3. Effects of acute and chronic pulsatile shear stress

**Figure 5.**
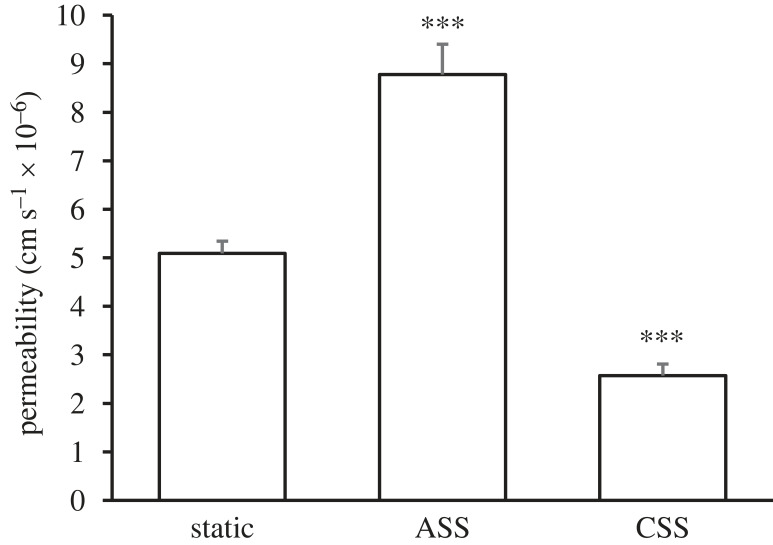
Effect of acute and chronic shear stress on monolayer permeability. Four hours of pulsatile flow (ASS) led to an increase in permeability whereas 3–5 days of pulsatile flow (CSS) led to a decrease in permeability, compared with permeability under static conditions. Mean + s.e.m.

Permeability was increased by acute shear stress (ASS; 8.78 ± 0.62 × 10^−6^ cm s^−1^; *n* = 14; *p* < 0.0001) and decreased by CSS (2.57 ± 0.24 × 10^−6^ cm s^−1^; *n* = 9; *p* < 0.0001) compared with static conditions (5.09 ± 0.25 × 10^−6^ cm s^−1^; *n* = 13) (figure 5).

### 3.4. Effect of increased viscosity

**Figure 6.**
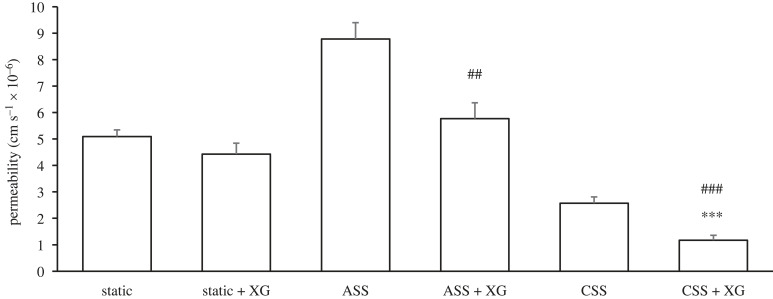
Effect of increased viscosity on monolayer permeability under static, ASS and CSS conditions. Xanthan gum (XG) was used to elevate shear stress, but not shear rate, under both ASS and CSS. In bioreactors perfused with medium containing XG, permeability was not significantly increased by acute shear stress (ASS) compared with static conditions, although there was a trend in that direction, while CSS produced a significant decrease. Equivalent data from the bioreactors perfused with conventional medium plus supplements are shown again for comparison. There was no effect of increased viscosity under static conditions, whereas the presence of XG appeared to lower permeability under both ASS and CSS. Mean + s.e.m. * significance of ASS + XG and CSS + XG data compared with static + XG; # significance of + XG data compared with the same flow condition without XG.

Four hours of pulsatile flow (ASS) with viscosity-increasing xanthan gum (XG) led to an increase in permeability of borderline significance (5.77 ± 0.60 × 10^−6^ cm s^−1^; 0.1 > *p* > 0.05) and 3–5 days of pulsatile flow (CSS) with XG led to a significant decrease in permeability (1.17 ± 0.19 × 10^−6^ cm s^−1^; *n* = 11; *p* < 0.0001), compared with static conditions with XG (4.43 ± 0.41 × 10^−6^ cm s^−1^; *n* = 7) (figure 6). Earlier data obtained under the same conditions but without XG are shown for comparison. Permeability was significantly higher under ASS without XG (*n* = 14) than with it (*n* = 7; *p* < 0.01). The same trend was observed under CSS: permeability values were higher without XG (*n* = 9) than with it (*n* = 11; *p* < 0.001). Under static conditions, there was no significant difference in permeability with (*n* = 7) and without XG (*n* = 13; *p* = NS). Permeability was thus affected under both acute and chronic shear by altering viscosity and hence shear stress, even though the shear rate remained unchanged.

### 3.5. Effects of flow waveform

**Figure 7.**
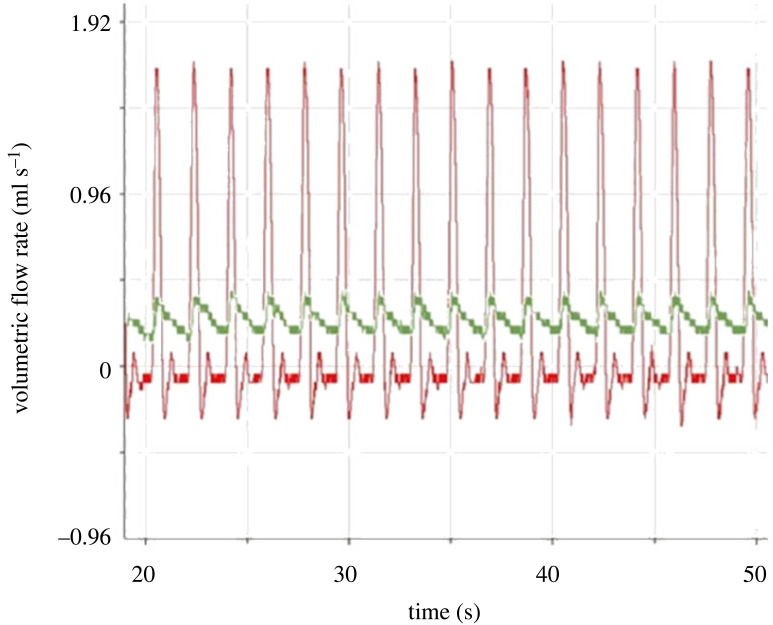
Control and damped flow waveforms. Flow waveform, determined with a transit-time ultrasound flow probe, under normal perfusion (red line) or with four 50 ml air-filled syringes attached to the bioreactor circuit to provide damping (green line). Peaks were aligned manually.

Figure 7 shows flow measured with zero and four 50 ml air-filled syringes attached to the bioreactor circuit. Flow without these damping chambers qualitatively resembled physiological flow in the aorta, although there were quantitative differences: there was forward-going flow over approximately one third of the cycle, followed by a period of reverse flow having smaller amplitude, and then a period of low amplitude forward-going flow. Each of these three periods had close to a triangular waveform. Finally, there was a period of constant, approximately zero flow. The fundamental frequency was 0.55 Hz. The amplitude of the waveform was approximately 2 ml s^−1^ and the mean flow was 0.25 ml s^−1^. Damping with four syringes reduced the amplitude to approximately 0.16 ml s^−1^; due to the use of a positive displacement pump, the mean flow and the fundamental frequency will have remained constant.

**Figure 8.**
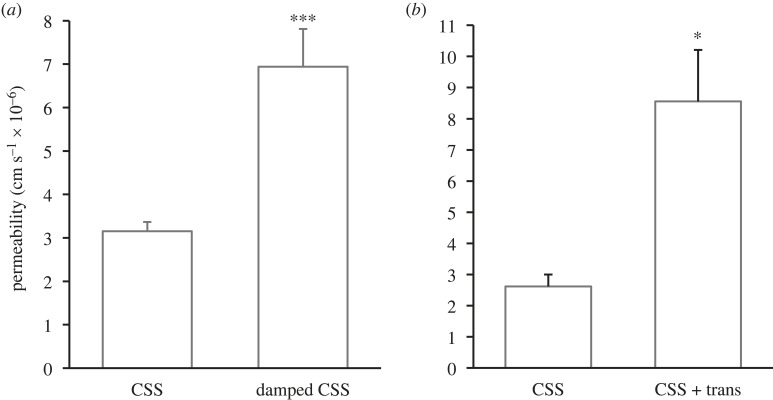
Effect of flow waveform and transmural flow on monolayer permeability. (*a*) Chronic damped pulsatile flow significantly increased permeability compared with the chronic normal pulsatile flow (CSS). (*b*) The addition of transmural flow (CSS + trans) also caused a significant increase in effective permeability compared with CSS alone. Mean ± s.e.m.

There was a significant increase in permeability when cells were chronically exposed to damped pulsatile flow (6.94 ± 0.87 × 10^−6^ cm s^−1^; *n* = 15; *p* < 0.0001) rather than the usual pulsatile flow (3.15 ± 0.218 × 10^−6^ cm s^−1^; *n* = 24) (figure 8*a*).

### 3.6. Effects of transmural flow

Flow from the intracapillary to the extracapillary space averaged approximately 0.1 ml h^−1^, which will have had a negligible effect on the rate of chronic pulsatile flow along the capillary lumen. The transmural flow was driven by the same pulsatile pressure gradient as the luminal flow and would there have had an oscillatory component.

Adding transmural flow to the normal chronic pulsatile flow (CSS) significantly increased effective permeability (8.56 ± 1.65 × 10^−6^ cm s^−1^; *p* < 0.05; *n* = 15) compared with chronic pulsatile flow without transmural flow (2.62 ± 0.38 × 10^−6^ cm s^−1^; *n* = 8) (figure 8*b*). The former is termed ‘effective permeability’ as it had a convective component.

A flow of 0.1 ml h^−1^ across the capillary surface area of 70 cm^2^ is equivalent to a convective mass transfer coefficient of 0.4 × 10^−6^ cm s^−1^, which is approximately 7% of the difference between these two results, so it could not directly account for the increase in albumin transport even if the reflection coefficient for albumin were zero. In practice, such coefficients are approximately 0.7 in cultured endothelial monolayers subject to an applied pressure difference, even in the absence of axial flow [28]. Hence we conclude that the increase in albumin transport is instead due to an increase in the true permeability of the monolayer induced by chronic transmural flow.

## 4. Discussion

The present study examined mechanical influences on the permeability of vascular endothelium *in vitro*. Aortic endothelial cells were used because the relation between stresses and permeability in large arteries is relevant to the patchy anatomical distribution of atherosclerosis. They were taken from pigs rather than a smaller species because there is allometric scaling of WSS by body weight [29,30]; WSS normally experienced by pig arterial endothelium should be similar to that seen by human cells.

Permeability was assessed by measuring the transport of albumin, a macromolecule that is widely used in such studies because it is present in plasma at high concentration, making it easy to detect, and because it is relatively inert. Receptor-mediated vesicular transport of albumin has been observed [31] but passive transport through normal or leaky intercellular junctions is thought to predominate for arterial endothelium [32]. Transport of the larger lipoproteins thought to be relevant to atherogenesis may [32] or may not [11,33] dominantly occur by the same route.

The cells were confluent and in a quiescent state before interventions commenced, as judged by measurements of permeability and glucose consumption, and remained confluent according to SEM images.

### 4.1. Effects of thrombin and L-NAME

The performance of the system, including the confluence of the monolayers, was checked by examining the effects of adding thrombin or L-NAME, which are known to increase permeability [34–36]. If there had been frequent or large gaps between cells of the monolayer, such behaviour would not be detectable: gaps are expected to dominate transport even if only 1 cell in 400 is missing [37]. Thrombin and L-NAME both altered permeability in the expected direction under chronic shear and the effects were large: permeability doubled with L-NAME and more than doubled with thrombin. We can therefore say that the monolayer in the hollow-fibre bioreactor is sufficiently confluent to test influences of other biological or mechanical agents, where effects are unknown, and can speculate that the monolayer has few defects.

### 4.2. Effects of acute and chronic pulsatile shear stress

Acute and chronic application of shear stress (ASS or CSS) have been shown to increase and decrease permeability, respectively, in the swirling well model when compared with static conditions [38]. Flow in that model is complex, being multidirectional as well as pulsatile [39–43]. Furthermore, mean shear, pulsatility and multidirectionality vary from the centre to the edge of the well, as does the influence of acute and chronic exposure to shear on permeability [44]. The present study is the first to compare ASS and CSS in a system where the shear is unidirectional and has spatially uniform mean magnitude and pulsatility; the interpretation of the results is consequently more straightforward.

ASS increased permeability to 1.7 × that seen under static conditions whereas CSS halved it. These values are essentially identical to those obtained in the swirling well model [38], indicating that they do not depend on the presence of complex flow but are a basic property of endothelium exposed to the type of flow expected in straight, unbranched arteries. The fact that the endothelial barrier is tightened by chronic flow with this physiological character is intuitively more satisfying than the opposite result, obtained in the acute experiments.

### 4.3. Effect of viscosity

Culture media used in most *in vitro* studies of endothelial permeability have a viscosity at 37°C of approximately 1 mPa s. By contrast, the value for blood is approximately 4 mPa s, depending on haematocrit. Furthermore, blood is a non-Newtonian, shear-thinning fluid whereas culture media are not. Obtaining physiological shear stresses at physiological shear rates therefore requires the addition of substances that increase viscosity, ideally in a non-Newtonian fashion, and a comparison of endothelial behaviour under flow with or without such additives can distinguish between responses to shear stress and shear rate.

Van den Broek *et al.* [27] have shown that the food additive xanthan gum is well suited for the purpose. Medium containing only 0.69 g l^−1^ of this bacterial polysaccharide approximates the viscosity and the shear-thinning behaviour of blood and is well tolerated by endothelial cells in prolonged culture; furthermore, the additive is stable under prolonged flow in *in vitro* systems [27]. The low mass concentration coupled with a high molecular weight (approximately 2 MDa) mean that only a small increment in osmotic pressure is produced.

Since the present study used a positive displacement pump, the mean flow rate will not have been affected by the change in viscosity. The flow was pulsatile and hence alteration of viscosity could theoretically have altered the time-varying velocity profiles occurring within the capillaries. However, the Womersley number for our configuration is less than 0.7 even without the xanthan gum; that is well within the viscous regime, so the flow would have been quasi-steady and velocity profiles (and therefore shear rates) should not have been affected by the additive.

Xanthan gum had no influence on permeability under static conditions, consistent with a lack of direct effect on endothelial cells or on transport. (The large size of the molecule means that it will be significantly excluded from the intercellular junction and hence will probably not slow diffusion through the cleft.) On the other hand, it significantly decreased permeability under both ASS and CSS, leading to a lack of significant difference between ASS and static conditions. These effects are attributable to altered shear stress and not to shear rate, which remained unchanged.

The normal effect of CSS—which is to reduce permeability—became larger when WSS was increased. However, the normal effect of ASS, which is to increase permeability, became smaller. We speculate that this is because the increase and then decrease in permeability after the imposition of shear is faster, as well as larger, when the magnitude of WSS is increased. Hence by 4 h, when transport under ASS was measured, permeability in the presence of the additive had dropped below the value seen without the additive.

Consistent with this speculation, Tarbell *et al.* [10] reported a comparable phenomenon for albumin transport when flow was applied and then stopped 60 min later. With both low and high applied shears, permeability increased when shear was applied and decreased when it ceased. At the higher shear, the initial increase in permeability was faster as well as larger than at the lower shear, but the return to baseline was also faster, leading to substantially (up to three-fold) *lower* permeability in endothelium that had been exposed to the higher shear between 30 and 90 mins after the cessation of shear.

### 4.4. Effects of flow waveform

As just noted, flow waveform varies from the centre to the edge of the swirling well. We [11,36,44] and others [45] have found differences in permeability from the centre to the edge, using specialized methods that give spatially resolved measurements of tracer or ion transport. However, mean shear and directionality also vary with radial distance, making it impossible to attribute the altered permeability solely to changes in flow waveform. In the present study, pulsatility was altered while mean magnitude and the unidirectional nature of the flow were kept constant, so such an attribution is possible.

The pulsatility of the flow produced by the standard pump was damped by incorporating air chambers into the circuit. Four 50 ml syringes reduced flow rate amplitude greater than 10-fold for the same mean flow: the ratio of amplitude to mean flow rate for the damped flow was approximately 10% of that seen in the human aorta [46], for example. Chronic exposure of endothelium to this damped pulsatile flow resulted in higher permeability than exposure to undamped pulsatile flow. Note that pulsatile flow is the norm in large arteries. Flow only becomes quasi-steady on progression through small arteries and arterioles.

Colgan *et al*. [47] drew the opposite conclusion, that steady flow gives lower permeability than pulsatile flow, from a comparison of orbital shaker (swirling well) and hollow-fibre bioreactor experiments. However, this inference was reached by assuming flow produced in the swirling well is steady. Numerical and experimental studies [39–43] contradict that assumption. Indeed, the flow may be more pulsatile than in the hollow-fibre bioreactor and will certainly be less uniaxial. The authors also acknowledge that cells were replated between flow exposure on the orbital shaker and the measurement of permeability, introducing an additional difference between the ‘steady’ and pulsatile conditions. Adding damping chambers to the hollow-fibre bioreactor provides a more controlled method.

If all endothelium behaves in the same way, then our data suggest that the endothelial barrier may be tighter in regions of highly pulsatile flow than in regions of less pulsatile flow (e.g. in arteries versus the microcirculation), and also that within arteries, areas experiencing highly pulsatile flow, perhaps due to geometric influences, might have locally tighter barriers.

### 4.5. Effects of transmural flow

Fluid transport across blood vessel walls occurs *in vivo* as a result of transmural gradients in oncotic and hydrostatic pressure; it advects solutes carried within plasma. The large majority of *in vitro* studies have not been able to include transmural flow for technical reasons, but it can be included when using the hollow-fibre bioreactor simply by removing the constant-volume constraint on the extracapillary space, and hence lowering the hydrostatic pressure on the abluminal side of the capillary.

Previous studies by Tarbell and co-workers showed that applying [48] or increasing [47] a pressure difference across an endothelial monolayer produces a sequence of at least three changes in transmural flow: there is an initial, effectively instantaneous increase from the baseline value, followed by a reduction in flow to a lower value, still above baseline, over 30–120 min—the so-called ‘sealing effect’—and then a further increase in flow which plateaus after a period of approximately 5 h. Our measurements were made after applying a pressure difference for substantially longer than 5 h.

Transmural flow gives rise to a fluid dynamic shear stress on the endothelial cell membrane within intercellular junctions. In our system, the transmural flow velocity (*J_v_*/*S*, where *J_v_* is the volume flux) averaged 4 × 10^−7^ cm s^−1^. Tarbell *et al*. [49] obtained a 10-fold higher baseline value of 3–4 × 10^−6^ cm s^−1^, and used two idealized models to show that the shear stress imposed on the walls of the cleft was of the order of 2.5–5.0 Pa [49]. In both models, shear was proportional to *J_v_*, so we can assume values of approximately 0.25–0.5 Pa in our experiments, which is comparable in magnitude to the wall shear stress on the luminal surface of 0.375 Pa. Tarbell *et al*. also estimated that the area of membrane in the clefts is 18% of the luminal membrane area. The substantial shear magnitude and large area over which it acts clearly could affect cell signalling. Transmural flow might thus alter monolayer properties through biological effects as well as by directly increasing solute transport through advection.

The addition of chronic transendothelial flow to chronic axial flow in the bioreactor increased transendothelial transport of rhodamine-labelled albumin more than could be explained by the addition of advection itself, even under the most favourable assumptions for the latter (i.e. a reflection coefficient of zero). The effect cannot be attributed to stretch because the capillary wall is rigid. We therefore conclude that transmural flow increased the permeability of the monolayer through a biological influence of junctional shear. Note that the effect is in the opposite direction to that of chronic luminal shear, which decreases permeability.

We did not examine the effect on albumin transport of short-term application of transmural flow but that has been investigated by DeMaio *et al*. [48], who measured albumin transport for 1 h before and 4 h after a pressure difference was applied across a monolayer. The sealing effect lasted approximately 2 h, during which period *J_v_* fell by approximately 70%. The effective permeability to albumin increased three-fold immediately after pressure was applied, but this increase was only approximately one third of the average transmural flow velocity (*J_v_*/*A*) and it decreased approximately in step with the latter over the sealing period; these data do not provide strong evidence for a flow-driven, biologically mediated increase in permeability.

The discrepancy with our own data may reflect differences in the experimental conditions, such as the absence of flow parallel to the endothelial surface or the application of a steady rather than a pulsatile pressure difference in the experiments of the Tarbell group. The discrepancy is also consistent with a difference between effects of acute and chronic transmural flow, as with luminal flow; chronic effects presumably have greater physiological relevance.

## 5. Conclusion

The unmodified hollow-fibre bioreactor is an excellent platform for examining effects of mechanical stresses on permeability, and its scope is increased by simple modifications that enable alteration of flow waveform and the imposition of both luminal and transendothelial flow. (The advantages and disadvantages of these systems is considered further in electronic supplementary material.) Using such devices, we have obtained novel data on the effects of acute versus chronic luminal flow, undamped versus damped pulsatile flow, shear rate versus shear stress and the absence or presence of flow though intercellular junctions.

## Data Availability

Data are available from: https://spiral.imperial.ac.uk/handle/10044/1/100942. The data are provided in electronic supplementary material [50].
